# P21 Overexpression Promotes Cell Death and Induces Senescence in Human Glioblastoma

**DOI:** 10.3390/cancers15041279

**Published:** 2023-02-17

**Authors:** Moustafa A. Mansour, Masum Rahman, Ahmad A. Ayad, Arthur E. Warrington, Terry C. Burns

**Affiliations:** 1 Department of Neurologic Surgery, Mayo Clinic, Rochester, MN 55905, USA; 2 Department of Neurologic Surgery, Faculty of Medicine, Al-Azhar University, Cairo 11884, Egypt

**Keywords:** P21, CDKN1A, glioblastoma, senescence, cancer senescence, gene overexpression, gene knock-in, CRISPR/Cas9, dCas, dCas-VPR

## Abstract

**Simple Summary:**

Current standard care for high-grade glioma involves maximal safe resection followed by chemoradiation. Recent studies showed that surviving cancer cells with an initially senescent phenotype following chemoradiation could escape senescence over time, giving rise to tumor cells that were more aggressive and resilient. Therefore, more powerful approaches are needed to either keep these glioma-initiating cells in a senescent state for longer or to eliminate these senescent cells prior to tumor recurrence. In this study, we demonstrate that P21 overexpression induces high levels of apoptosis in multiple human glioma cell lines and, in surviving cells, promotes cell cycle arrest and senescent gene expression. Additionally, we demonstrate that P21 overexpression induces senescence more rapidly and stably than the irradiation of human glioblastoma cells. Finally, we find that P21-overexpressing glioma cells selectively depend upon Bcl-xL to avoid apoptotic cell death.

**Abstract:**

High-grade gliomas are the most common and aggressive adult primary brain tumors with a median survival of only 12–15 months. Current standard therapy consists of maximal safe surgical resection followed by DNA-damaging agents, such as irradiation and chemotherapy that can delay but not prevent inevitable recurrence. Some have interpreted glioma recurrence as evidence of glioma stem cells which persist in a relatively quiescent state after irradiation and chemotherapy, before the ultimate cell cycle re-entry and glioma recurrence. Conversely, latent cancer cells with a therapy-induced senescent phenotype have been shown to escape senescence, giving rise to more aggressive stem-like tumor cells than those present in the original tumor. Therefore, approaches are needed to either eliminate or keep these glioma initiating cells in a senescent state for a longer time to prolong survival. In our current study, we demonstrate that the radiation-induced cell cycle inhibitor P21 can provide a powerful route to induce cell death in short-term explants of PDXs derived from three molecularly diverse human gliomas. Additionally, cells not killed by P21 overexpression were maintained in a stable senescent state for longer than control cells. Collectively, these data suggest that P21 activation may provide an attractive therapeutic target to improve therapeutic outcomes.

## 1. Introduction

Senescence is the process by which cells stop dividing and enter a state of growth arrest without undergoing cell death. Cellular senescence is a key component of human aging that can be induced by unrepaired DNA damage or other cellular stresses [1,2]. Senescence is a key mechanism of tumor suppression that may be mediated by the DNA damage response or via key oncogenes, such as ras, cyclin E, raf, and E2F3 [3,4,5]. The tumor-suppressive impact of senescence may result from directly inhibiting tumor proliferation or by stimulating an immune response against malignant cells. The role of senescence in tumor suppression is supported in part by a higher percentage of senescent cells in pre-malignant lesions than malignant lesions, suggesting a role of senescence in blocking cancer progression [6,7,8]. Glioblastoma (GBM) is a fatal infiltrative brain tumor for which standard treatment includes maximal surgical resection followed by irradiation and alkylating chemotherapy, typically with temozolomide (TMZ) [9]. Unfortunately, recurrence is inevitable, suggesting the failure of irradiation and TMZ to maintain senescence in glioblastoma cells. Therefore, avenues to either eliminate senescent cells after therapy or prevent senescent escape could improve outcomes.

Activation of the p53/P21^WAF1/CIP1^ and p16^INK4A^/pRB tumor suppressor pathways plays a central role in regulating senescence in normal mammalian cells, but cancer cells dysregulate these genes to maintain their malignant features of continuous proliferation and invasion [10]. For example, the homozygous deletion of CDKN2A, which encodes p16, is pathognomonic for increased aggressiveness in glioma and other cancers [11,12]. P21 is a potent cyclin-dependent kinase inhibitor (CKI) that binds to and inhibits the activity of cyclin-CDK2, -CDK1, and -CDK4/6 complexes, and thus functions as a regulator of cell cycle progression at the G1 and S phases [13,14]. Although cell cycle arrest is a cardinal feature of senescence, whether externally inducing cell cycle arrest can induce other features of senescence is unclear. Although P21 is a well-characterized marker of senescence, the mechanistic role of P21 in glioblastoma survival and therapy-induced senescence has not been rigorously evaluated. P21 has been reported to have paradoxically cancer-promoting impacts in certain contexts. Here, we demonstrate that P21 overexpression induces high levels of apoptosis in multiple human glioma cells lines, and in surviving cells, promotes cell cycle arrest and senescent gene expression. Conversely, P21 downregulation promotes survival and proliferation. Additionally, we demonstrate that P21 overexpression induces senescence more rapidly and stably than the irradiation of human glioblastoma cells. As previously demonstrated with radiation-induced senescence, we find that P21-overexpressing glioma cells are selectively dependent upon Bcl-xL to avoid apoptotic cell death.

## 2. Results

### 2.1. Radiation Induces Senescent-like Gene Expression in a Dose-Dependent Manner

**Figure 1:**
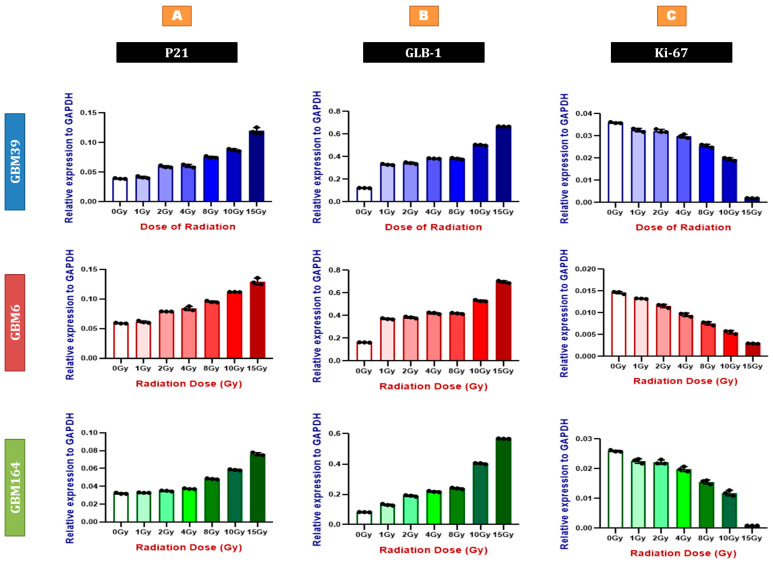
Radiation induces senescent-like gene expression in a dose-dependent manner. qRT-PCR for P21 (**A**), GLB-1 (**B**), and ki-67 (**C**) using RNA extracted from GBM39 (**Blue**), GBM6 (**Red**), and GBM164 (**Green**) cells after different doses of irradiation, as shown in the Materials and Methods compared to the sham (0 Gy) irradiated group. Y axes demonstrate expression relative to the house-keeping gene, GAPDH. Individual data points are shown, representing a biological triplicate of 3 independent experiments. Each data point represents a mean of a technical triplicate. Error bars represent SD between the independent experiments.

Radiation is well known to induce senescence and P21 upregulation. To determine if the level of P21 expression correlates with radiation-induced senescence in a dose-dependent manner, we used three human glioblastoma cell lines with divergent molecular features (Table 1). Each cell line was treated with varying doses of radiation from 0 to 15 Gy. mRNA was isolated and used to evaluate the gene expression of P21, GLB-1 (senescence-specific beta-galactosidase), and ki-67 (proliferation marker) (Figure 1 and Figure S1). We found that both P21 and GLB-1 expression positively correlate with radiation dose (Figure 1A,B and Figure S1A,B), while ki-67 inversely correlates with radiation dose (Figure 1A,C and Figure S1A,C), suggesting a positive correlation between P21 and senescence, and a negative correlation between P21 and proliferation.

### 2.2. P21 Overexpression Inhibits Human GBM Proliferation and Promotes Cellular Senescence

**Figure 2:**
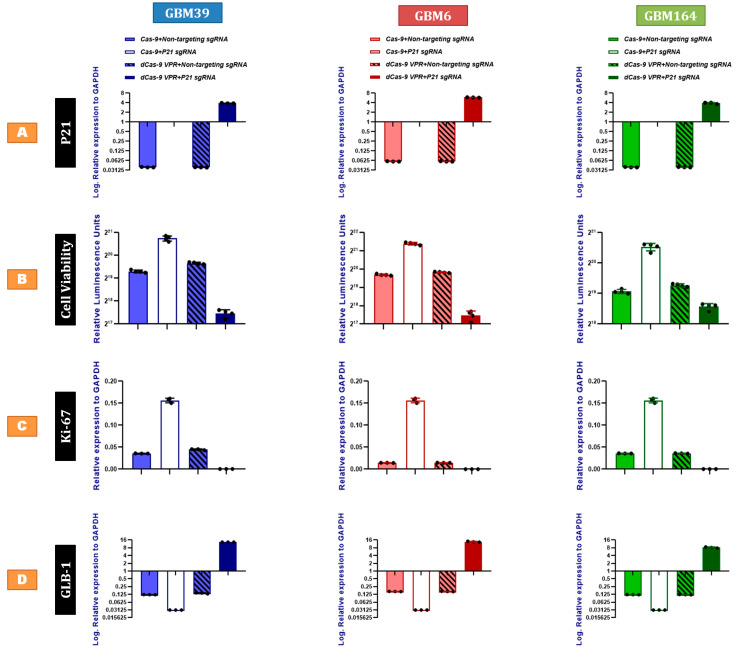
P21 overexpression inhibits human GBM proliferation and promotes cellular senescence. (**A**) qRT-PCR for P21 confirming the gene editing (knock-out using CRISPR/Cas9 and knock-in using dCas9-VPR) compared to the corresponding controls treated with non-targeting sgRNAs, using RNA extracted from GBM cells, as shown including GBM39 (**Blue**), GBM6 (**Red**), and GBM164 (**Green**). Y axes (**A**,**C**,**D**) demonstrate log expression relative to the house-keeping gene, GAPDH. (**B**) Cell titer-glo assay demonstrating the cell viability of GBM cells in response to the knock-in vs. the knock-out of P21, compared to the same cells treated with scrambled non-targeting sgRNAs. (**C**) qRT-PCR for ki-67 in the P21-genetically modified cells (P21 knock-in vs. P21 knock-out) compared to the corresponding controls treated with non-targeting sgRNAs. (**D**) qRT-PCR for GLB-1 in the P21-genetically modified cells (P21 knock-in vs. P21 knock-out) compared to the corresponding controls treated with non-targeting sgRNAs. Results were obtained from three independent experiments. Each data point represents the mean of technical triplicates. Error bars represent SD between the independent experiments, *p* value < 0.0001.

To functionally determine how P21 expression impacts measures of senescence and viability in glioma cells, we utilized CRISPR-based gain and loss of function strategies to modulate P21 expression. We utilized CRISPR/dCas-VPR and CRISPR/Cas9 technology to overexpress and delete P21, respectively, in each cell line using non-targeting sgRNA sequences as controls for each experiment. The cells were evaluated after genetic modification for cell viability and gene expression. The resultant decline and gain of P21 expression were evaluated by qRT-PCR (Figure 2A) and Western blotting (Figure S2). In each line, P21-deficient cultures (Figure 2B) yielded significantly higher viability, while P21-overexpressing cells showed lower viability, as evaluated via an ATP-mediated luminescent assay. This correlated with a significant increase in ki-67 expression and decreased expression of GLB-1 levels with P21 loss, while the opposite was true with P21 overexpression (Figure 2C,D). Collectively, these data suggested that P21 may act through the regulation of both cell proliferation and viability.

### 2.3. P21 Overexpression Induces Tumor Cell Apoptosis by Activating the p53-Upregulated Modulator of Apoptosis

**Figure 3:**
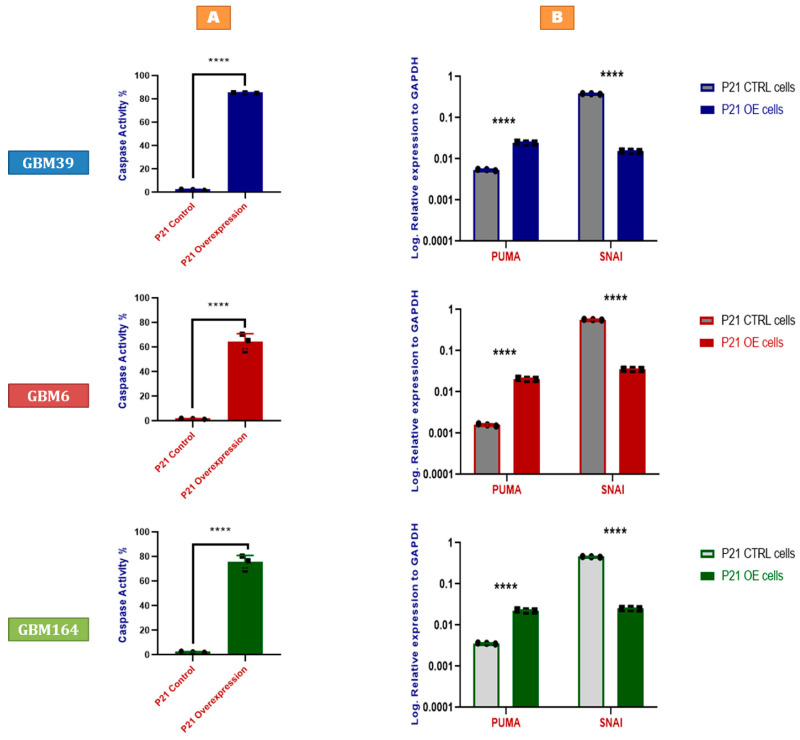
P21 overexpression induces tumor cell apoptosis by activating the p53-upregulated modulator of apoptosis. (**A**) Fluorescence-based caspase assay indicating caspase-3 enzymatic activity in GBM39 (**Blue**), GBM6 (**Red**), and GBM164 (**Green**) cells in response to P21 knock-in (overexpression). Values are normalized to results acquired from the same cell lines treated with scrambled non-targeting sgRNAs. Error bars represent SD from three different samples (biological triplicates), as indicated by the individually plotted data points. Each data point represents the mean of three technical replicates. (**B**) qRT-PCR for PUMA and SNAI (SLUG) in P21-overexpressing (knock-in) cells compared to the corresponding control cells treated with a non-targeting scrambled sgRNA, using RNA extracted from GBM39 (**Blue**), GBM6 (**Red**), and GBM164 (**Green**) cells. Y axes demonstrate log expression relative to the house-keeping gene, GAPDH. Individual data points are shown, representing a biological triplicate of 3 independent experiments. Each data point represents a mean of a technical triplicate. Error bars represent SD between the independent experiments. A summary of the *p* value is shown [**** *p* values < 0.0001].

The inverse correlation between ATP level and P21 expression could reflect more rapid proliferation in the P21-deficient cells; however, the early timepoint of analysis after genetic alteration makes this unlikely to be the sole explanation. As such, we also asked if P21 activity could also directly impact cell viability, independent of cell proliferation, via an evaluation of caspase-3 activity. Indeed, P21 overexpression increased caspase-3 enzymatic activity, suggesting that P21 induction directly promotes apoptosis (Figure 3A). The critical role of P21 in cellular senescence is well recognized. Our prior work suggested that PUMA (p53-upregulated modulator of apoptosis) promotes apoptosis in senescent glioma cells but is countered by Bcl-xL to enable survival. Similarly, we found increased PUMA expression in each line of P21-overexpressing human GBM (Figure 3B), which likely facilitates apoptosis in response to P21 overexpression. Indeed, PUMA mRNA expression demonstrates a positive correlation with P21 across patients in the TCGA data set (Figure S3A). Furthermore, SNAI (SLUG), a well-known biological inhibitor of PUMA and p53 [16], was significantly decreased in P21-overexpressing cells for each GBM line, positioning SNAI as a plausible regulator of P21-mediated PUMA expression (Figure 3B). The RNA and protein co-expression analysis of TCGA GBM data via cBioPortal revealed positively correlated mRNA and protein co-expression of CDKN1A. and BAX (Figure S3B,C). To test if the upregulation of P21 directly upregulates BAX, we performed a Western blot analysis for BAX in GBM6 (P53-mutant) and GBM39 (P53-WT), and observed markedly increased expression (Figure S3D), consistent with a BAX-mediated downstream mechanism of cell death in response to P21 overexpression.

### 2.4. P21-Mediated Senescence in GBM Cells

**Figure 4:**
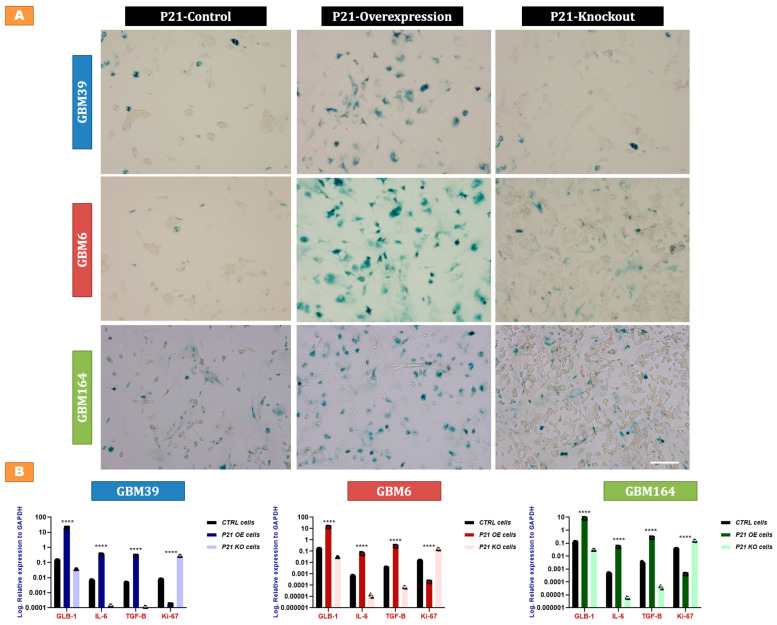
P21-mediated senescence in GBM cells. (**A**) Senescence-associated beta-galactosidase staining in the P21-genetically modified (P21 knock-in vs. P21 knock-out) GBM39 (**Blue**), GBM6 (**Red**), and GBM164 (**Green**) cells, compared to the corresponding control cells treated with non-targeting sgRNAs. Scale bar = 100 µm. (**B**) qRT-PCR for GLB-1, IL-6, TGF-B, and ki-67 in the P21-genetically modified cells (P21 knock-in vs. P21 knock-out) compared to the corresponding control cells treated with non-targeting sgRNAs using RNA extracted from GBM39 (**Blue**), GBM6 (**Red**), and GBM164 (**Green**) cells. Y axes demonstrate log expression relative to the house-keeping gene, GAPDH. Individual data points are shown, representing a biological triplicate of 3 independent experiments. Each data point represents a mean of a technical triplicate. Error bars represent SD between the independent experiments. A summary of the *p* value is shown [**** *p* values < 0.0001].

Since some cells survived direct P21 overexpression, we sought to further characterize these surviving cells. Our earlier experiments (Figure 2C,D) demonstrated decreased proliferation and increased GLB-1 in P21-overexpressing cells, which was suggestive of senescence. As such, we asked if such cells also demonstrate histologic features of senescence, including enzymatic beta-galactosidase (beta-gal) activity and the expression of senescence-associated secretory phenotype (SASP) [17,18]. P21-deleted cells demonstrated no significant change in the baseline low percentage of cells with beta galactosidase staining, though all three lines showed markedly increased beta-gal staining upon P21 overexpression (Figure 4A). Further consistent with a senescent phenotype, we also observed an increased expression of the SASP factors, transforming growth factor beta (TGF-β), interleukin 6 (IL-6), and galactosidase beta 1 (GLB-1), along with a marked drop in the nuclear protein (ki-67) in P21-overexpressing cells, while opposite findings were detected in P21-deleted cells (Figure 4B).

### 2.5. Faster and More Stable Senescence with P21 Overexpression than Irradiation

**Figure 5:**
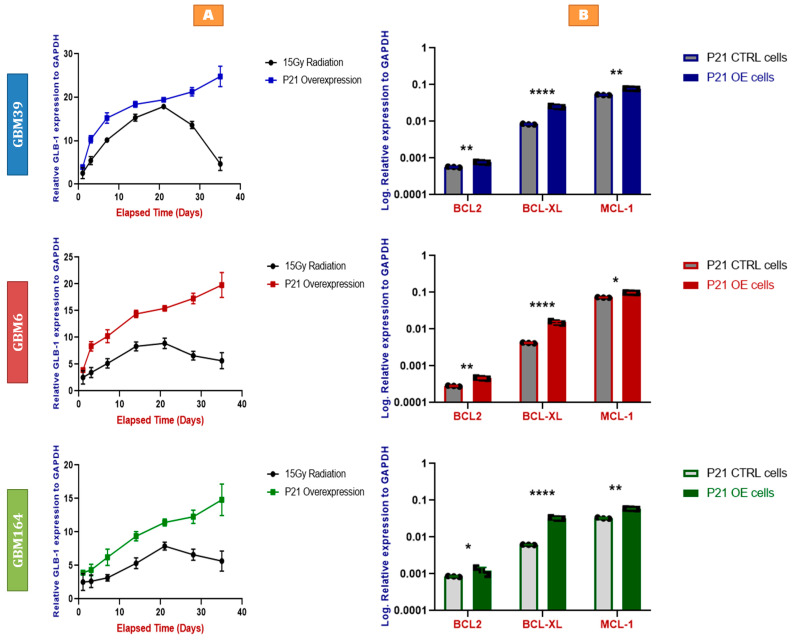
(**A**) Faster and more stable senescence with P21 overexpression than irradiation. qRT-PCR for GLB-1 in the P21-overexpressing (knock-in) cells compared to 15Gy-irradiated cells, using RNA extracted from GBM39 (**Blue**), GBM6 (**Red**), and GBM164 (**Green**) cells at different time points (1, 3, 7, 14, 21, 28, and 35 days). Y axes demonstrate log expression relative to the house-keeping gene, GAPDH. Individual data points are shown; each data point represents a biological triplicate of 3 independent experiments. Error bars represent SD between the independent experiments (biological triplicate). (**B**) P21-induced senescence relies on Bcl-xL for survival. qRT-PCR for BCL2, Bcl-xL, and MCL-1 in P21-overexpressing (knock-in) cells compared to the corresponding control cells treated with a non-targeting scrambled sgRNA, using RNA extracted from GBM39 (**Blue**), GBM6 (**Red**), and GBM164 (**Green**) cells. Y axes demonstrate log expression relative to the house-keeping gene, GAPDH. Individual data points are shown, representing a biological triplicate of 3 independent experiments. Each data point represents a mean of a technical triplicate. Error bars represent SD between the independent experiments. A summary of the *p* values is shown [* *p* values < 0.05, ** *p* values < 0.01, **** *p* values < 0.0001].

Senescence can be induced by DNA damaging agents, including radiation, which is one of the standard therapies against glioblastoma. Unfortunately, glioblastoma escapes senescence, as evidenced via inevitable recurrence [19]. To assess the relative impacts of radiation- versus P21 overexpression-induced senescence, we treated each of the three cell lines with P21-overexpressing vectors versus 15 Gy radiation, which induces senescence in human glioblastoma cells [19]. Interestingly, we observed a more rapid elevation and a stable maintenance of GLB-1 levels in P21-overexpressing cells compared to irradiation-treated cells, wherein GLB-1 induction was less robust and started to decline after 21 days (Figure 5A).

### 2.6. P21-Induced Senescence Relies on Bcl-xL for Survival

We have previously shown that chemotherapy and radiation-induced GBM cells escape apoptosis via the anti-apoptotic actions of Bcl-xL. We asked if Bcl-xL could also be relevant to cell survival in the context of direct P21-induced senescence. Seven days after P21 overexpression, we observed an upregulation of multiple anti-apoptotic protein-coding genes, including BCL2, Bcl-xL, and MCL-1 (Figure 5B). The fold-change from baseline in these cells was greatest for Bcl-xL, suggesting a potentially increased dependency of these human glioma cells upon Bcl-xL after P21 exposure.

### 2.7. Bcl-xL Knock-Down Induces Apoptosis in P21-Induced Senescent Glioblastoma Cells

**Figure 6:**
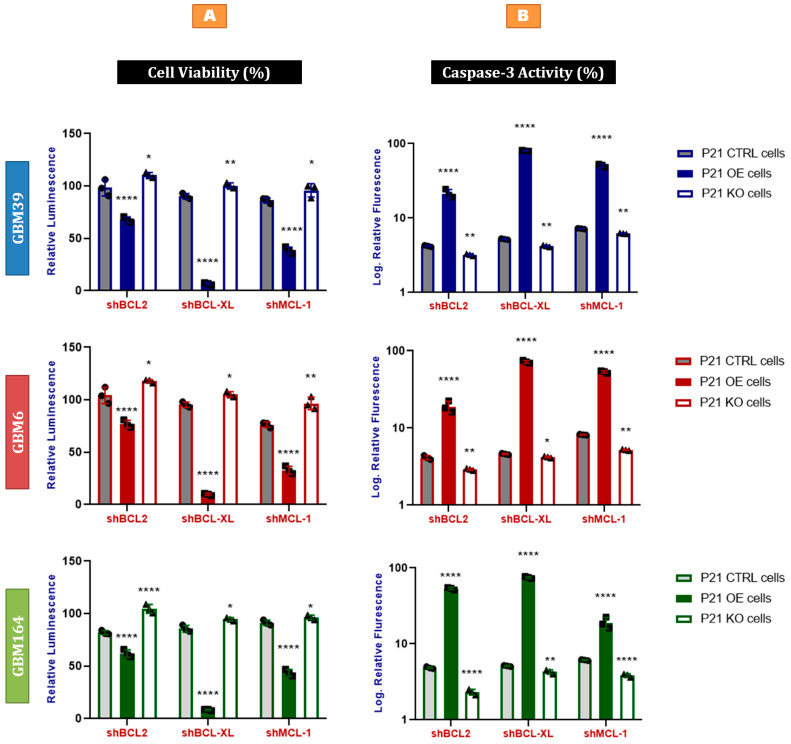
Bcl-xL knock-down induces apoptosis in P21-induced senescent glioblastoma cells. (**A**) Cell titer-glo assay demonstrating the cell viability of GBM39 (**Blue**), GBM6 (**Red**), and GBM164 (**Green**) P21-genetically modified cells (knock-in and knock-out) compared to the corresponding control cells, in response to the knock-down of BCL2, Bcl-xL, and MCL-1, using shBCL2, shBcl-xL, and shMCL-1 vectors. Error bars represent SD values from three different samples (biological triplicates) of each cell line, as indicated by the individually plotted data points. Each data point represents the mean of three technical replicates. (**B**) Fluorescence-based caspase assay demonstrating the caspase-3 activity in GBM39 (**Blue**), GBM6 (**Red**), and GBM164 (**Green**) P21-genetically modified cells (knock-in and knock-out) compared to the corresponding control cells, in response to the knock-down of BCL2, Bcl-xL, and MCL-1, using shBCL2, shBcl-xL, and shMCL-1 vectors. Error bars represent SD values from three different samples (biological triplicates) of each cell line, as indicated by the individually plotted data points. Each data point represents the mean of three technical replicates. A summary of *p* values is shown [* *p* values < 0.05, ** *p* values < 0.01, **** *p* values < 0.0001].

Based on these data, we used shRNAs targeting BCL2, Bcl-xL, and MCL-1 anti-apoptotic proteins in stably P21-overexpressing senescent glioblastoma cells, as compared to naïve cells or cells deficient in P21. By 3 days after BCL2, Bcl-xL, or MCL-1 knock-down, we observed a significant drop in cell viability of P21-overexpressing cells, with most marked decline in cells receiving the Bcl-xL knock-down (Figure 6A). These data are similar to our prior findings of Bcl-xL dependency in TMZ- and radiation-induced senescent GBM cells [19], and could suggest that induced Bcl-xL dependency is mediated through P21. Consistent with our previously reported pro-apoptotic mechanism of PUMA-mediated cell death upon Bcl-xL blockade, we also detected a significant increase in caspase-3 enzymatic activity in each p21-overexpressing cell line in response to Bcl-xL knock-down (Figure 6B). Conversely, applying these shRNAs to P21-control or P21-deficient glioblastoma cells yielded relatively minimal impact on cell viability (Figure 6).

## 3. Methods

### 3.1. Cell Culture and Growth Conditions

GBM6, GBM39, and GBM164 human glioblastoma cell lines were obtained from the National (Patient-Derived Xenografts) PDX resource [15]. All cell lines were cultured and maintained in antibiotic-free FBS media, composed of 10% Fetal Bovine Serum (FBS) and 90% Dulbecco’s Modified Eagle’s medium (DMEM). The cells were passaged regularly once they reached 70–90% confluence using phosphate-buffered saline (PBS) and 1X Trypsin-EDTA (0.05% Trypsin and 0.53mM of EDTA). All experiments were performed using third passage cultured cells.

### 3.2. Irradiation of Cells

One to three × 10^6^ GBM cells were plated either in 10 cm or 7 cm of sterile tissue culture vessels and maintained in culture for 2–3 days until they became 50–60% confluent; then, they were exposed to cesium γ-radiation (1, 2, 4, 8, 10, or 15Gy).

### 3.3. Cell Viability Assay

The number of viable cells in culture was measured using the Cell Titer-Glo^®^ Luminescent Cell Viability Assay, as per the manufacturer’s instructions and protocol (Promega, G7570, G7571, G7572, and G7573, Madison, WI, USA). The Cell Titer-Glo^®^ Luminescent Cell Viability Assay is a method to determine the number of viable cells in culture based on a quantification of ATP to detect metabolically active cells.

### 3.4. Caspase-3 Assay

Caspase-3 enzymatic activity was measured in cell lysates prepared from GBM cell lines using the Caspase-3 Activity Assay Kit (Cell Signaling Technology, #5723, Danvers, MA, USA) as per the manufacturer’s instructions. The Caspase-3 Activity Assay Kit is a fluorescent assay that detects caspase-3 in cell lysates. It contains a fluorogenic substrate (N-Acetyl-Asp-Glu-Val-Asp-7-amino-4-methylcoumarin or Ac-DEVD-AMC) for caspase-3. During the assay, activated caspase-3 cleaves this substrate between DEVD and AMC, generating highly fluorescent AMC that can be detected using a fluorescence reader with excitation at 380 nm and emission between 420 and 460 nm. Cleavage of the substrate only occurs in the lysates of apoptotic cells. Therefore, the amount of AMC produced is proportional to the number of apoptotic cells in the sample.

### 3.5. Quantitative Real-Time PCR (qRT-PCR)

RNA was extracted from the GBM cells as follows. The cells were washed with PBS before being homogenized with TRIzol reagent (Invitrogen, Carlsbad CA, USA). RNA precipitation was performed at −20 °C overnight (12–18 h). The precipitated RNA pellets were dissolved in RNase-free water, and the concentration was measured by absorbance at 260 nm (A260) using Nanodrop2000. cDNA synthesis was performed with 1 µg of total RNA using a M-MLV reverse transcriptase kit (ThermoFisher, # 28025013 and 28025021, Waltham, MA, USA), as per the manufacturer’s protocol. An amount of 25 ng of cDNA was used for the real-time PCR by TaqMan gene expression assay targeting the gene of interest on an ABI 7500/7500-Fast Real-Time PCR System (Applied Bioscience, Beverly Hills, CA, USA). The relative expression of each gene was determined by the ΔΔCT method.

### 3.6. Viral Packaging and Gene Knock-Down by shRNA

Plasmid sequences for shRNAs were either designed manually using commercially available software or purchased as already designed sequences from Addgene. The sequence was provided in the form of a bacterial plasmid. Bacteria were cultured in lysogeny broth (LB) media and ampicillin (to kill any intervening bacteria without the plasmid of interest). A single bacterial colony was selected from the bacterial culture and further expanded. Plasmid DNA from the bacteria was isolated and purified using the PureLink™ HiPure Plasmid Miniprep kit (Invitrogen, #K210002 and K210003), as per the manufacturer’s protocol. Isolated DNA concentration and purification were measured using NanoDrop (ThermoFisher, Waltham, MA, USA). Viral packaging was performed as per the manufacturer’s (Addgene, Watertown, MA, USA) protocol using 10 µg of DNA plasmid to transfect 3.8 × 10^6^ HEK293 cells in a 10 cm tissue culture dish. The virus was harvested at 72 h post-transfection. Viral supernatant was centrifuged at 500 *g* for 5 min to pellet any packaging cells that were collected during harvesting. The supernatant was filtered through a 0.45 µm polyethersulfone (PES) filter. Viral supernatant was aliquoted, snap-frozen in liquid nitrogen, and stored at −80 °C to avoid loss until time transduction was performed. Five to eight × 10^5^ GBM cells were plated in six-well plates and incubated at 37 °C overnight (16–18 h). The following day, 10–20 µL of viral supernatant was added to each well and incubated at 37 °C for 36–72 h. The protein was then extracted to confirm knock-down, and the cells were re-plated for any further experiments.

### 3.7. Gene Knock-Out by CRISPR/Cas9

Plasmids encoding for Cas9 protein were purchased from the Addgene online service along with plasmids encoding for the puromycin resistance gene. The provided bacterial plasmids were cultured in lysogeny broth (LB) media and ampicillin. A single bacterial colony was selected from the bacterial culture and further expanded. DNA plasmid from the bacteria was isolated and purified using a PureLink™ HiPure Plasmid Miniprep kit (Invitrogen, #K210002 and K210003), as per the manufacturer’s protocol. Isolated DNA concentration and purification were assayed using NanoDrop. Viral packaging was performed as described in the viral packaging section. GBM cells were cultured in a six-well plate at a density of 5–8 × 10^5^ cells in 2 mL of antibiotic-free media/well. The cells were incubated overnight (16–18 h) at 37 °C. The following day, 10–20 µL of viral supernatant was added to each well and incubated at 37 °C for 48–72 h. The transduced cells then were treated with puromycin for 48 h to ablate all non-transduced cells. The surviving cells were expanded in culture. The presence of Cas9 in the cells was verified by Western blot using a Guide-it Cas9 polyclonal antibody (TaKaRa, Cat. Nos. 632606 & 632607, San Jose, CA, USA). Viral vector encoding for P21 was generated using the same method as described above. Cas9 gene editing activity and the success of the CRISPR knock-out were checked using the Guide-it Mutation Detection kit (TaKaRa, Cat. No. 631448), followed by another step of confirmation by qRT-PCR and Western blotting. By using qRT-PCR, the knocked-out P21 was undetectable, even at high RNA concentrations, confirming the success of CRISPR/Cas9 knock-out.

### 3.8. Gene Knock-In (Overexpression) by dCas-VPR

The protocol can be divided into 2 parts:

#### 3.8.1. Generating Cell Lines That Constitutively Express dCas9-VPR

GBM cells were plated overnight in six-well plates with the optimum cell density of 8 × 10^5^ cells per well in reduced-serum medium (Optimum Medium). The following day, the cells were transduced with lentiviral particles carrying dead or deactivated cas9 (dCas9) fused with one or more transcriptional activators (dCas9-VPR) along with blasticidin resistance marker (BlastR) (Dharmacon, Lafayette, CO, USA). The transduction performed with a mode of infection (MOI) equals 0.3 transduction units per cell. On the third day post-transduction, the media were changed and replaced with selection media containing blasticidin antibiotic (selection media). The selection media were replaced after 2 days with antibiotic-free media. The cells were kept in the antibiotic-free media for expansion, while some cells were harvested from the culture to confirm the expression of dCas9-VPR within them by Western blotting.

#### 3.8.2. Inducing P21 Overexpression

The dCas9-expressing GBM cells were plated overnight in six-well plates with a cellular density of 4 ×10^5^ cells per well. On the following day, 10–20 μL of viral supernatant encoding for P21 was added to each well of the six-well plate. Two days post-transduction, some cells were used for RNA extraction to confirm the gene overexpression by qRT-PCR. We optimized the number of cells and the viral supernatant volume mentioned above after several trials to ensure that all the cells would overexpress P21.

### 3.9. Senescence-Associated β-Galactosidase Staining

A senescence-associated β-galactosidase staining kit was used as an indicator of senescence after P21 overexpression, as per the manufacturer’s directions. Briefly, cells were fixed for 10 min in β-galactosidase fixative solution (10% 100 × fixative solution; 90% H_2_O) and washed with PBS. The cells were then stained with β-galactosidase staining solution (93% 1 × staining solution; 1% 100 × solution; 1% 100 × solution B; 5% X-gal). Wells without samples were filled with PBS, and the plate was wrapped in parafilm to prevent evaporation. The plate was left overnight in a dry incubator at 37 °C. The next day, the cells were examined under a microscope for β-gal-positive cells (blue staining).

### 3.10. Protein Analysis by Western Blotting (WB)

Cells grown in six-well plates, 10 cm dishes, or T-25 flasks were washed with PBS, dissociated in 1X Trypsin-EDTA, and collected in 1.5 micro-centrifuge tubes as a cell pellet. The cell pellet was then lysed using lysis buffer (10% RIPA lysis buffer; 4% protease-inhibitor cocktail; 1% phosphatase-inhibitor cocktail-2; 1% phosphatase-inhibitor cocktail-3; 84% molecular grade water). The cell pellet with the lysis buffer was then sonicated for 30 min in a water bath sonicator (one-minute sonication every other minute for a total of 30 min). The whole lysate was then centrifuged for 10 min at the speed of 17,000× *g* to collect the supernatant as the final protein lysate. The concentration of the final protein lysate was then measured using the BCA kit, with albumin as the standard protein. Proteins extracted from the cells using lysis buffer were separated in SDS-polyacrylamide gel electrophoresis along with the optimum protein ladder, using 4–12% Bis-Tris gel. The proteins were then transferred to a polyvinylidene difluoride (PVDF) membrane. The membrane was blocked in 5% fat-free milk for 30 min, washed three times (5 min each) using Tris-buffered saline with Tween20 (TBST), and then probed with the desired antibodies.

### 3.11. Statistical Analysis

All the experiments were repeated at least three times. The statistical analysis was performed using a one-way ANOVA and unpaired two-tailed *t*-test using GraphPad prism 8.4.2. Error bars represent the SD values and significance was taken as * *p* < 0.05, ** *p* < 0.01, *** *p* < 0.001, **** *p* < 0.0001. Unless otherwise stated, each data point reflects the average of three technical replicates. Error bars show the standard deviation of biological replicates.

### 3.12. Reagents and Antibodies

A list of the chemicals, reagents, and antibodies used in the study is provided below (Table 2).

## 4. Discussion

Recent studies have shown that surviving cancer cells with an initially senescent phenotype following chemoradiation were able to escape senescence over time, giving rise to tumor cells that were more aggressive and resilient than those present in the original tumor [20,21,22,23,24,25]. Therefore, more powerful approaches are needed to either keep these glioma initiating cells in a senescent state for longer, or to eliminate these senescent cells prior to tumor recurrence.

In our current study, we demonstrate that P21 is a potentially powerful mediator of cell fate able to single-handedly replicate or exceed the pro-apoptotic and senescence-inducing impacts of high-dose radiation.

P21 is a potent cell cycle inhibitor, which binds and inhibits several cyclins and cyclin-dependent kinases, such as CDK2, -CDK1, and -CDK4/6 complexes [13,14]. Cells with high P21 typically enter a G0/quiescent state, whilst those with low P21 continue to proliferate [26]. Since P21 is downstream to p53, which is frequently mutated in cancer, P21 activity can be dysregulated in cancer. Indeed, some studies have suggested that P21 can paradoxically promote proliferation in cancer cells under certain conditions [27,28,29,30,31]. We directly manipulated P21 in both P53-mutant (GBM6) and P53-WT cells with (GBM164) and without (GBM39) IDH-mutation and observed P21-induced apoptosis and senescence in surviving cells in each cell line.

P53 regulates both apoptosis and senescence. P21 is downstream of P53 and considered a master regulator of senescence, while other mechanisms are thought to regulate P53-mediated apoptosis. As such, the robust finding of the direct P21-mediated upregulation of apoptosis was unexpected. The pro-apoptotic gene PUMA is considered downstream of P53 rather than P21, although P53-indepenent regulation is well documented. Of note, PUMA and P53 transcription are both negatively regulated by SLUG, which was also downregulated in the P21 overexpressing cells and may help explain the observed PUMA upregulation in P21-overexpresing cells [16,23]. That PUMA was induced by P21 overexpression in GBM6, which lacks functional P53, suggests that P21 is sufficient to directly regulate SNAI/PUMA to impact cell survival in a P53-independent manner.

P21-induced senescence is consistent with prior reports. However, its powerful and lasting impact in GBM cells raises the question of whether P53-independent P21 activity could be leveraged directly for its anti-tumorigenic potential. Our data suggest that direct P21 activation, if possible, could help circumvent mutant P53 without compromising the ability to induce apoptosis or maintain senescence. Further studies on a broader array of P53-mutant cells will be necessary to determine whether this strategy could be more widely generalizable. Moreover, in vivo studies would be needed to determine if the pro-apoptotic and antiproliferative impacts of P21 induction we observed in vitro could translate into a survival advantage. If so, pharmacologic efforts aimed at direct P21 induction within cancer cells could provide a novel strategy for cancer control.

Although P21 overexpression induced cell death in a large percentage of glioma cells, some cells were able to survive, as is typical of cancer cells in response to almost any cancer therapy. These cells started to exhibit characteristic morphological features including increased size, new irregular shapes, a darker nucleus, cytoplasmic vacuolation, and other features characteristic of senescent cells [32]. Senescent phenotype was confirmed by beta-galactosidase staining and qRT-PCR for GLB-1, as well as SASP factors IL-6 and TGF-β [33,34,35,36,37,38]. Curiously and excitingly, direct P21 overexpression appeared to induce a senescent phenotype more rapidly, intensely, and in a more lasting manner compared to high-dose (15Gy) radiation, as evaluated via GLB-1 expression (Figure 5A).

We next asked which mechanisms may sustain the survival of these P21-induced senescent cells, since an ideal anti-cancer therapy would invoke not only senescent induction, but also the complete ablation of malignant cells. Our prior work demonstrated that senescent glioma cells are selectively dependent upon Bcl-xL, as compared to non-senescent cells [19]. Of the anti-apoptotic Bcl-2 family members, we found that P21 overexpression induced the most prominent upregulation of Bcl-xL, as compared to BCL2 and MCL-1 (Figure 5B). Functionally, P21-overexpressing cells were also relatively more vulnerable to caspase-3-mediated apoptosis following the depletion of Bcl-xL, as compared to BCL2 or MCL-1 (Figure 6A,B), which was consistent with our prior findings in radiation or TMZ-induced senescent cells [19].

It is worth noting that P21 (CDKN1A) mRNA co-expression analysis using human GBM TCGA data via cBioPortal reveals over 6000 significantly correlated genes (q value < 0.05). This is consistent with the substantial impacts of P21 as a master regulator of senescence. The highest ranked hallmark gene sets included P53 and apoptosis, followed by IFN-gamma response genes and MTORC1 signaling proteins, findings that are consistent with elevated BAX and mTOR proteins with P21 overexpression (Figures S3 and S4). More complete insights will require full transcriptome and proteomic analyses across a wider range of molecularly diverse GBMs. To determine if observed in vitro findings will be maintained in an in vivo setting, the induction of P21 will be required in situ within established gliomas—a challenge and opportunity for future studies.

## 5. Conclusions

Collectively, these findings demonstrate that P21 is a powerful mediator of glioma cell fate, which promotes desirable outcomes of apoptosis, as well as prolonged and sustained senescence in surviving cells. Identifying P53-independent avenues to directly upregulate P21 in situ within malignant cells may provide novel opportunities to improve outcomes across molecularly diverse gliomas.

## Figures and Tables

**Table 1:** Genetic and molecular characteristics of the 3 different GBM cell lines (GBM6, GBM39, and GBM164) used for the experiments shown in our current study. Data were generated and collected from the cBioPortal as in [15].

Sex	Male	Male	Female
Age	65	51	38
Recurrence Status	Primary	Primary	Primary
MGMT Methylation	U	M	M
Sub-type	Classical	Mesenchymal	Proneural
IDH-1	-	-	Mh
CDKN2A	LL	L	LL
PTEN	L	LM	L
EGFR	A (V3)	A (V3)	-
TP53	M	-	-
Met.	A	A	A
TERT Prom.	M	M	-
Others	-	MDM4 & PIKC32B (A)	PDGFR (A); NF1 (L); ATRX (T)

M = Mutant; A = Amplified or net gain (>2n); L = Loss; LL = Homozygous deletion; h = Heterozygous; V = Variant; T = Truncation.

**Table 2:** List of chemicals, reagents, and antibodies used in the study.

Reagent	Manufacturer	Catalogue Number
DMEM (Dulbecco’s Modified Eagle’s Medium)	Corning	10-013-CV
Trypsin EDTA 1X	Corning	25-052-CI
Opti-MEM™ I Reduced Serum Medium	Gibco	31-985-070
Blasticidin S HCl	ThermoFisher	A1113903
Puromycin Dihydrochloride	ThermoFisher	A1113803
Guide-it Cas9 Polyclonal Antibody	TaKaRa	632606 & 632607
Guide-it Mutation Detection kit	TaKaRa	631448
PureLink™ HiPure Plasmid Miniprep kit	Invitrogen	K210002 and K210003
LB Broth	Gibco	10855021
M-MLV reverse transcriptase kit	ThermoFisher	28025013 and 28025021
The Cell Titer-Glo^®^	Promega	G7570, G7571, G7572, and G7573
Caspase-3 Activity Assay Kit	Cell Signaling Technology (CST)	5723
TRIzol™ Reagent	Invitrogen	15596018
Senescence-associated β-Galactosidase Staining Kit	Cell Signaling Technology (CST)	9860
Pierce™ BCA Protein Assay Kit	ThermoFisher	23225
NuPAGE™ 4 to 12%, Bis-Tris, 1.0–1.5 mm, Mini Protein Gels	Invitrogen	NP0322BOX & NP0321BOX
Immun-Blot PVDF Membrane	Bio-Rad	1620177
Non-fat Dry Milk	Cell Signaling Technology (CST)	9999
P21 Antibody for WB	Cell Signaling Technology (CST)	2947
GAPDH	Cell Signaling Technology (CST)	8884

Designed sequences for primers, shRNAs, and sgRNAs used in this study are available upon request from the corresponding authors.

## Data Availability

Data supporting the findings of this study are available on request from the corresponding authors.

## Supplementary Materials

The following supporting information can be downloaded at: https://www.mdpi.com/article/10.3390/cancers15041279/s1, Figure S1. An existing correlation between P21 and senescence in human glioblastoma. Figure S2. Western blotting confirmation of the resultant knock-out and overexpression of P21 across the three cell lines used in this study. Figure S3. P21 positively correlates with PUMA and BAX using co-expression analysis. Figure S4. P21 inversely correlates with mTOR using co-expression analysis.
