# Supplementary material for: P21 Overexpression Promotes Cell Death and Induces Senescence in Human Glioblastoma
Source: Cancers (Basel). 2023 Feb 17;15(4):1279. doi: 10.3390/cancers15041279 (PMC9954583; doi:10.3390/cancers15041279)
Supplement: Supplementary file 1 [file cancers-15-01279-s001.zip › cancers-2143213-supplementary.pdf]

P21 Overexpression Promotes Cell Death and Induces Senescence in Human Glioblastoma

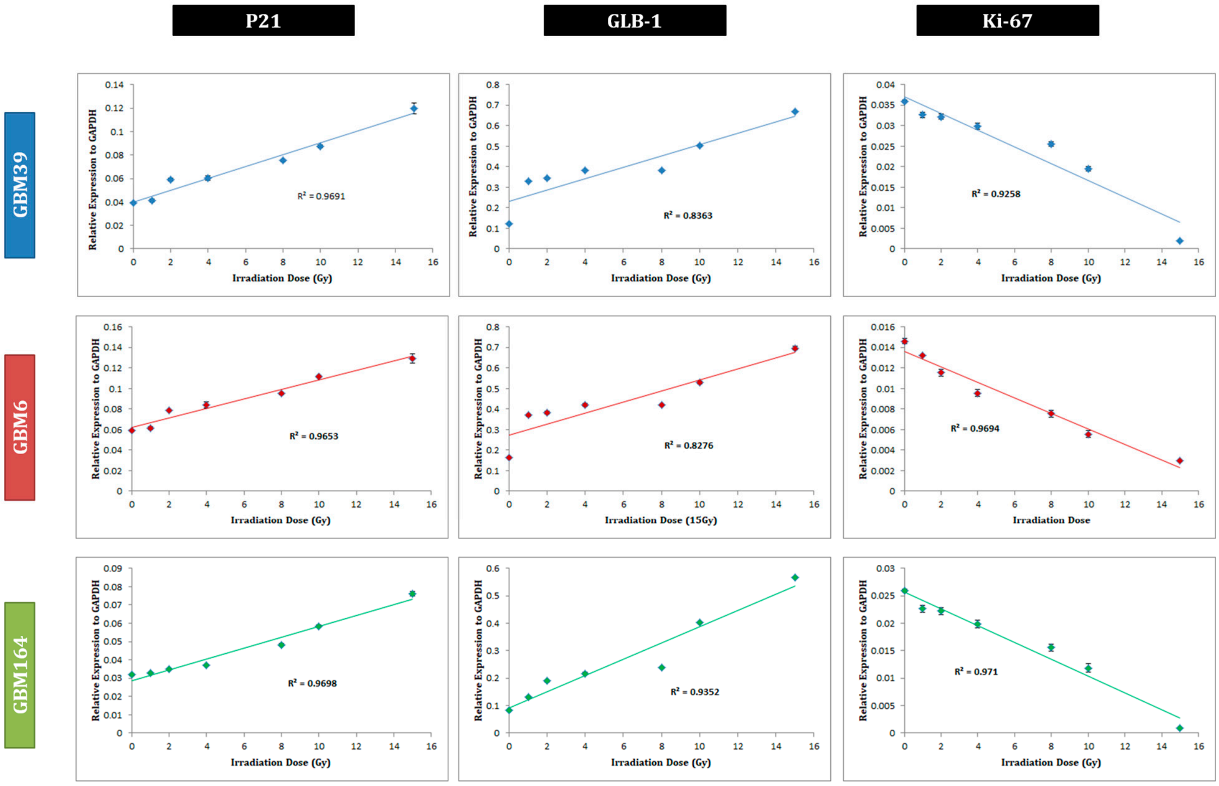

[**Figure S1**]: **An existing correlation between P21 and senescence in human glioblastoma.**

qRT-PCR for P21 (**A**), GLB-1 (**B**), and ki-67 (**C**) using RNA extracted from GBM39 (**Blue**), GBM6 (**Red**), and GBM164 (**Green**) cells after different doses of irradiation as shown in the materials and methods compared to the sham (0Gy) irradiated group. Y axes demonstrate expression relative to the house-keeping gene, GAPDH. Each data point represents a mean of a biological triplicate from three independent experiments. Error bars represent SD between the independent experiments. A linear correlation is shown with a displayed R^2.

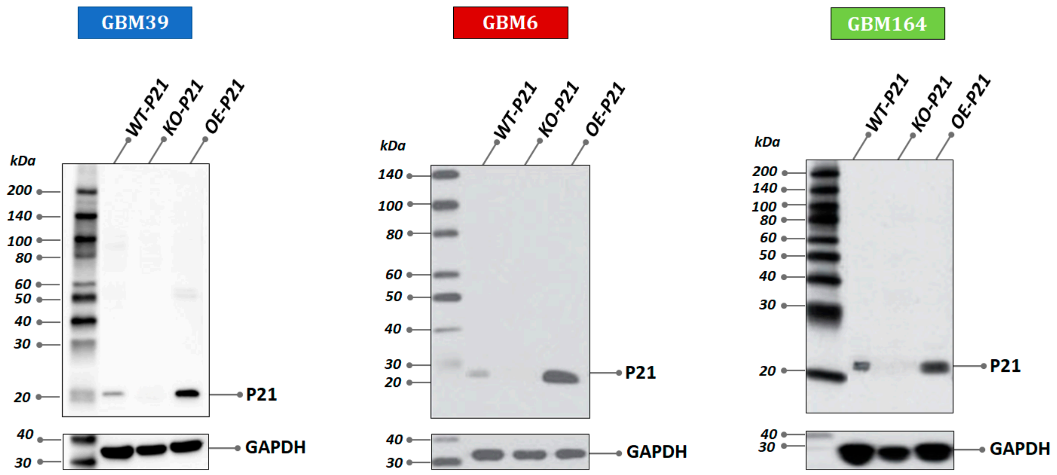

[Figure S2]: Western blotting confirmation of the resultant knock-out and overexpression of P21 across the three cell lines used in this study.

Western blotting for P21 using lysates from GBM39 (**Blue**), GBM6 (**Red**), and GBM164 (**Green**) cells after being treated with molecular vectors to either knock-out (KO) or overexpress (OE) P21. GAPDH was used as a loading control.

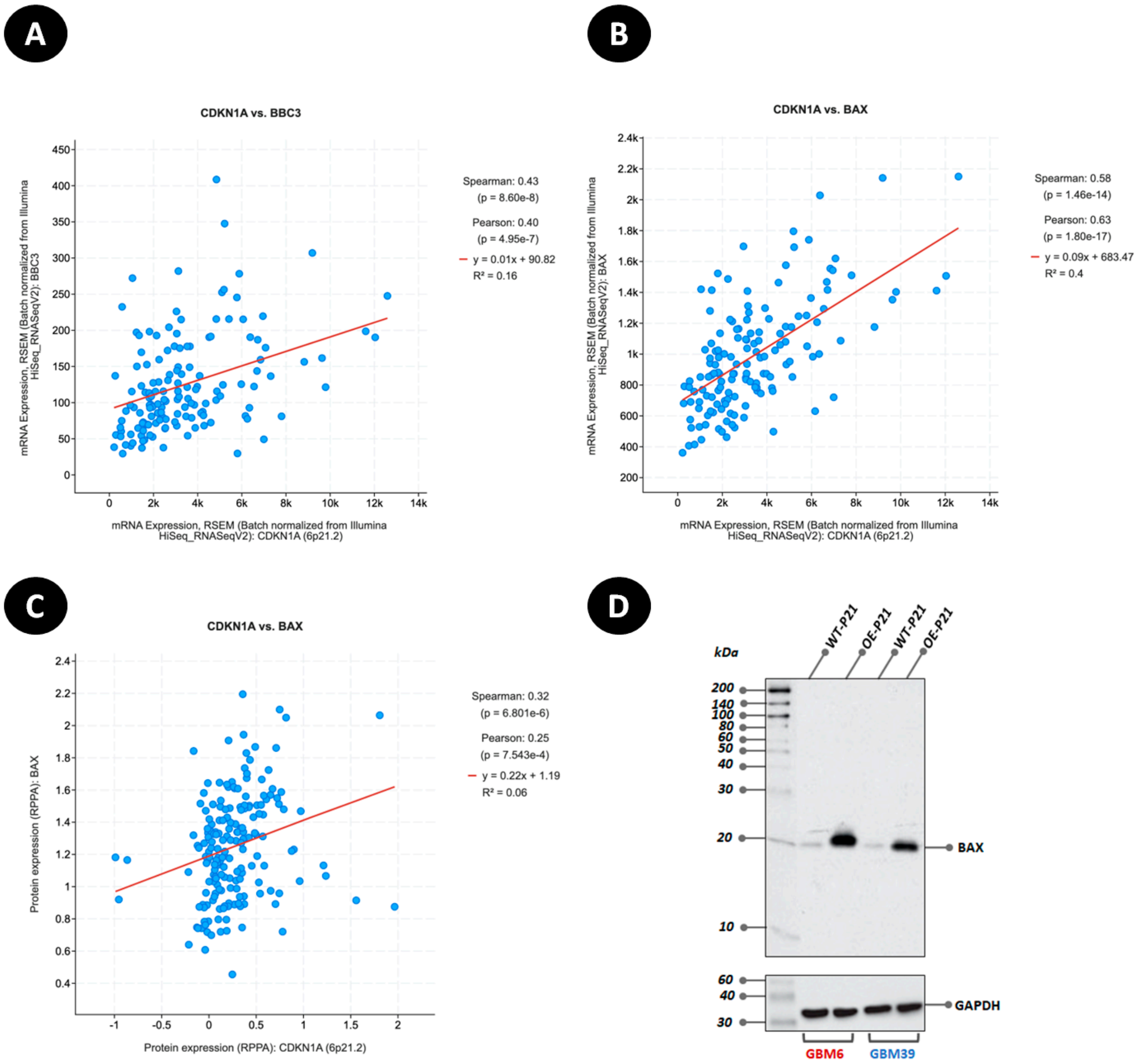

[Figure S3]: P21 positively correlates with PUMA and BAX using co-expression analysis.

(**A**) P21 (*also known as* CDKN1A) mRNA expression correlation analysis with PUMA (*also known as* BBC3) in human glioblastoma from the TCGA data set (PanCancer Atlas), using CBioPortal.org, demonstrating a positive co-expression correlation (Spearman correlation co-efficient=8.60e-8). P21 mRNA (**B**) and protein (**C**) expression correlation analysis with BAX in human glioblastoma from the TCGA data set (PanCancer Atlas), demonstrating positive co-expression correlations (Spearman correlation co-efficient=1.46e-14 and 6.801e-4, respectively). (**D**) Western blotting analysis of BAX protein expression in P21-wild type (WT) versus P21-overexpressing (OE) GBM6 and GBM39 cell lines demonstrating a higher upregulation in P21-overexpressing cells.

A

Gene Set Name [# Genes (K)]	Description	# Genes in Overlap (k)	k/K	p-value	FDRq-value
HALLMARK_P53_PATHWAY [200]	Genes involved in p53 pathways and networks.	15		$1.85\ e^{-18}$	$9.25\ e^{-17}$
HALLMARK_APOPTOSIS [161]	Genes mediating programmed cell death (apoptosis) by activation of caspases.	7		$1.39\ e^{-7}$	$3.46\ e^{-6}$
HALLMARK_INTERFERON_GAMMA_RESPONSE [200]	Genes up-regulated in response to IFNG [GeneID=3458].	5		$1.29\ e^{-4}$	$1.62\ e^{-3}$
HALLMARK_MTORC1_SIGNALING [200]	Genes up-regulated through activation of mTORC1 complex.	5		$1.29\ e^{-4}$	$1.62\ e^{-3}$
HALLMARK_CHOLESTEROL_HOMEOSTASIS [74]	Genes involved in cholesterol homeostasis.	3		$7.81\ e^{-4}$	$7.81\ e^{-3}$
HALLMARK_IL6_JAK_STAT3_SIGNALING [87]	Genes up-regulated by IL6 [GeneID=3569] via STAT3 [GeneID=6774], e.g., during acute phase response.	3		$1.25\ e^{-3}$	$7.99\ e^{-3}$
HALLMARK_COMPLEMENT [200]	Genes encoding components of the complement system, which is part of the innate immune system.	4		$1.44\ e^{-3}$	$7.99\ e^{-3}$
HALLMARK_HYPOXIA [200]	Genes up-regulated in response to low oxygen levels (hypoxia).	4		$1.44\ e^{-3}$	$7.99\ e^{-3}$
HALLMARK_TNFA_SIGNALING_VIA_NFKB [200]	Genes regulated by NF-kB in response to TNF [GeneID=7124].	4		$1.44\ e^{-3}$	$7.99\ e^{-3}$
HALLMARK_INTERFERON_ALPHA_RESPONSE [97]	Genes up-regulated in response to alpha interferon proteins.	3		$1.71\ e^{-3}$	$8.53\ e^{-3}$

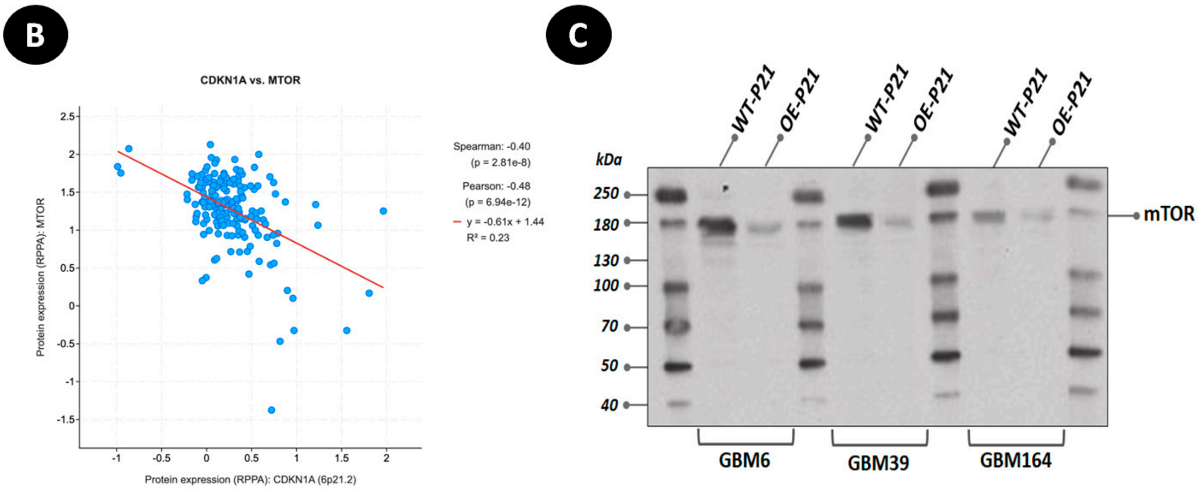

[**Figure S4**]: **P21 inversely correlates with mTOR using co-expression analysis.**

(**A**) P21 (CDKN1A) mRNA co-expression analysis using human glioblastoma TCGA data via cBioPortal revealing the highest ranked hallmark gene sets. (**B**) P21 (CDKN1A) protein expression correlation analysis with mTOR in human glioblastoma from the TCGA data set (PanCancer Atlas), using CBioPortal.org, demonstrating an inverse co-expression correlation (Spearman correlation co-efficient=2.81e-8). (**C**) Western blotting analysis of mTOR protein expression in P21-wild type (WT) versus P21-overexpressing (OE) GBM6,39,164 cell lines demonstrating a lower expression in P21-OE cells compared to P21-WT cells.
